# Exploring the Potential of Abexinostat as a Therapeutic Agent for Amyloid‐Beta Pathology in Alzheimer’s Disease: A Multimodal Approach

**DOI:** 10.1002/alz70859_096802

**Published:** 2025-12-25

**Authors:** Lalit Sharma, Anik Kumar Das

**Affiliations:** ^1^ Shoolini University, Solan, Himachal Pradesh India

## Abstract

**Background:**

Alzheimer’s disease (AD) is a progressive neurodegenerative disorder characterized by cognitive decline and neuronal damage, with amyloid‐beta (Aβ) aggregation being a hallmark of AD pathology. Histone deacetylase (HDAC) inhibitors, including Abexinostat, have shown potential in modulating neurodegenerative processes by influencing protein aggregation and oxidative stress. This study aimed to evaluate the therapeutic potential of Abexinostat in AD through computational, in vitro, and in vivo methodologies.

**Method:**

Computational docking studies assessed the binding interactions between Abexinostat and Aβ to explore its potential to inhibit Aβ aggregation. In vitro experiments evaluated the effects of Abexinostat on Aβ42 fibril formation, protease digestion, and oxidative stress modulation. Western blotting measured the expression of key proteins involved in Aβ metabolism, including Aβ precursor protein (APP) and beta‐secretase (BACE1). RT‐PCR analysis examined the expression of antioxidant genes, such as superoxide dismutase (SOD) and catalase. Cognitive function and memory were evaluated in an Aβ42 intracerebroventricular (ICV) mouse model using the Morris water maze and Y‐maze. Histopathological analysis assessed amyloid plaque burden and neuronal damage in brain sections.

**Results:**

Computational docking studies revealed strong binding between Abexinostat and Aβ, indicating its potential to inhibit Aβ aggregation. In vitro, Abexinostat reduced Aβ42 fibril formation by 45% at 10 µM and 72% at 20 µM (p < 0.05, p < 0.01) and promoted protease digestion by 33% at 10 µM and 58% at 20 µM (p < 0.01). Western blotting showed a 34% reduction in APP and a 42% reduction in BACE1 expression (p < 0.05, p < 0.01). RT‐PCR analysis demonstrated a 52% increase in SOD and a 39% increase in catalase expression (p < 0.01, p < 0.05). Behavioral tests showed a 40% reduction in escape latency and a 35% increase in platform crossings in the Morris water maze (p < 0.05, p < 0.01). Histopathological analysis revealed a 50% reduction in amyloid plaque burden and 41% less neuronal damage (p < 0.01, p < 0.05).

**Conclusion:**

Abexinostat demonstrates potential as a dual‐target therapeutic for AD, inhibiting Aβ aggregation and improving cognitive function by modulating oxidative stress and Aβ metabolism. Further investigation is needed to explore its clinical applications.

